# The Covert World of Fish Biofluorescence: A Phylogenetically Widespread and Phenotypically Variable Phenomenon

**DOI:** 10.1371/journal.pone.0083259

**Published:** 2014-01-08

**Authors:** John S. Sparks, Robert C. Schelly, W. Leo Smith, Matthew P. Davis, Dan Tchernov, Vincent A. Pieribone, David F. Gruber

**Affiliations:** 1 Department of Ichthyology, American Museum of Natural History, Division of Vertebrate Zoology, New York, New York United States of America; 2 Sackler Institute for Comparative Genomics, American Museum of Natural History, New York, New York, United States of America; 3 Biodiversity Institute, University of Kansas, Lawrence, Kansas, United States of America; 4 Marine Biology Department, The Leon H. Charney School of Marine Sciences, University of Haifa, Mount Carmel, Haifa, Israel; 5 Department of Cellular and Molecular Physiology, The John B. Pierce Laboratory, Inc., Yale University, New Haven, Connecticut, United States of America; 6 Department of Natural Sciences, Baruch College, City University of New York, New York, New York, United States of America; Consiglio Nazionale delle Ricerche (CNR), Italy

## Abstract

The discovery of fluorescent proteins has revolutionized experimental biology. Whereas the majority of fluorescent proteins have been identified from cnidarians, recently several fluorescent proteins have been isolated across the animal tree of life. Here we show that biofluorescence is not only phylogenetically widespread, but is also phenotypically variable across both cartilaginous and bony fishes, highlighting its evolutionary history and the possibility for discovery of numerous novel fluorescent proteins. Fish biofluorescence is especially common and morphologically variable in cryptically patterned coral-reef lineages. We identified 16 orders, 50 families, 105 genera, and more than 180 species of biofluorescent fishes. We have also reconstructed our current understanding of the phylogenetic distribution of biofluorescence for ray-finned fishes. The presence of yellow long-pass intraocular filters in many biofluorescent fish lineages and the substantive color vision capabilities of coral-reef fishes suggest that they are capable of detecting fluoresced light. We present species-specific emission patterns among closely related species, indicating that biofluorescence potentially functions in intraspecific communication and evidence that fluorescence can be used for camouflage. This research provides insight into the distribution, evolution, and phenotypic variability of biofluorescence in marine lineages and examines the role this variation may play.

## Introduction

The primarily monochromatic blue spectrum that characterizes large areas of the photic ocean provides a unique filtered-light environment for visual organisms. Compared to the terrestrial environment, marine organisms reside in a spectrally restricted visual domain. The red, orange, yellow, and green components of sunlight are selectively removed with depth resulting in a narrow, near-monochromatic, band of blue light between 470 and 480 nm [1]. Spectrally restricted illumination in the ocean provides unique lighting conditions for organisms to exploit fluorescence to produce visual contrast and patterns. In the marine environment, biofluorescence is highly prevalent in cnidarians (particularly Anthozoans) [2], and also in a ctenophore [3], copepods [4], mantis shrimp [5], amphioxus [6] and some fishes [7]. In addition, the photosynthetic apparatus associated with chlorophyll fluoresces red and provides a background of biofluorescence in areas of high algal growth on coral reefs.

Biofluorescence results from the absorption of electromagnetic radiation at one wavelength by an organism, followed by its reemission at a longer and lower energy wavelength, visually resulting in green, orange, and red emission coloration in marine organisms. Biofluorescence signaling has previously been reported in butterflies [7], parrots [9], spiders [10], and flowers [11], as well as a deep-sea siphonophore [12]. In scleractinian corals, biofluorescence has been suggested to function in photoprotection [13], antioxidation [14], regulation of symbiotic dinoflagellates [15], photoacclimation [16], visual contrast [2], and coral health [17].

Whereas insight into the evolution and function of biofluorescence has greatly enhanced our knowledge of coral biology, little to nothing is known regarding the impact of biofluorescence on other organisms that thrive in coral-reef habitats, particularly those with advanced visual systems that could readily exploit fluorescent coloration and contrast. Investigating the evolution of biofluorescence across marine fishes is particularly appealing because they are visual animals, many of which possess yellow intraocular (lenses or cornea) filters [18], which function as long-pass filters and could enable enhanced perception of biofluorescence in the ocean. Worldwide, there are more than 8,000 species of fishes that inhabit coral reefs. Many reef fish species are known for their striking color patterns, whereas many others are cryptically patterned and appear well camouflaged. However, nearly nothing is known regarding the evolution or function of fluorescence in fishes. Only recently has a fluorescent protein, a novel fatty-acid-binding protein, been isolated from a vertebrate, a Japanese eel [19].

Here we report, for the first time, that biofluorescence is widespread throughout the tree of life for fishes, and it appears particularly common and phenotypically variable in marine lineages, especially cryptically patterned, well camouflaged coral-reef lineages. Our findings identify a widespread and previously unrecognized evolutionary phenomenon that provides new insights into the evolution of marine fishes and the function of light and visual systems in a marine environment, as well as providing a framework for the discovery of additional novel fluorescent proteins.

## Methods

Research, collecting and export permits were obtained from the government of the Bahamas, from the Ministry of Fisheries and Ministry of Environment, Honiara, Solomon Islands, and from the Department of Environment, Cayman Islands Government. This study was carried out in strict accordance with the recommendations in the Guidelines for the Use of Fishes in Research of the American Fisheries Society and the American Museum of Natural History's Institutional Animal Care and Use Committee (IACUC). Fishes were collected via SCUBA, using both standard open circuit systems and closed circuit rebreathers, via the application of rotenone and quinaldine to a targeted variety of shallow to deep (mesophotic) habitats in each sampling location where collecting was permitted.

Taxonomic field surveys of biofluorescence in marine fishes were conducted during the following expeditions: Little Cayman Island, January 2011, working out of the Central Caribbean Marine Institute; the Exumas, Bahamas, May 2011 and December 2011, at the Perry Institute for Marine Science on Lee Stocking Island; and a taxonomically comprehensive survey conducted at numerous localities in the Solomon Islands (June–July, 2012 and September 2013). In addition, we have supplemented these field studies with specimens available in the aquarium trade and by imaging specimens at aquariums after hours (e.g., Mystic Aquarium and Institute for Exploration, Mystic, CT; Birch Aquarium, Scripps Institution of Oceanography, La Jolla, CA).

**Figure 1:**
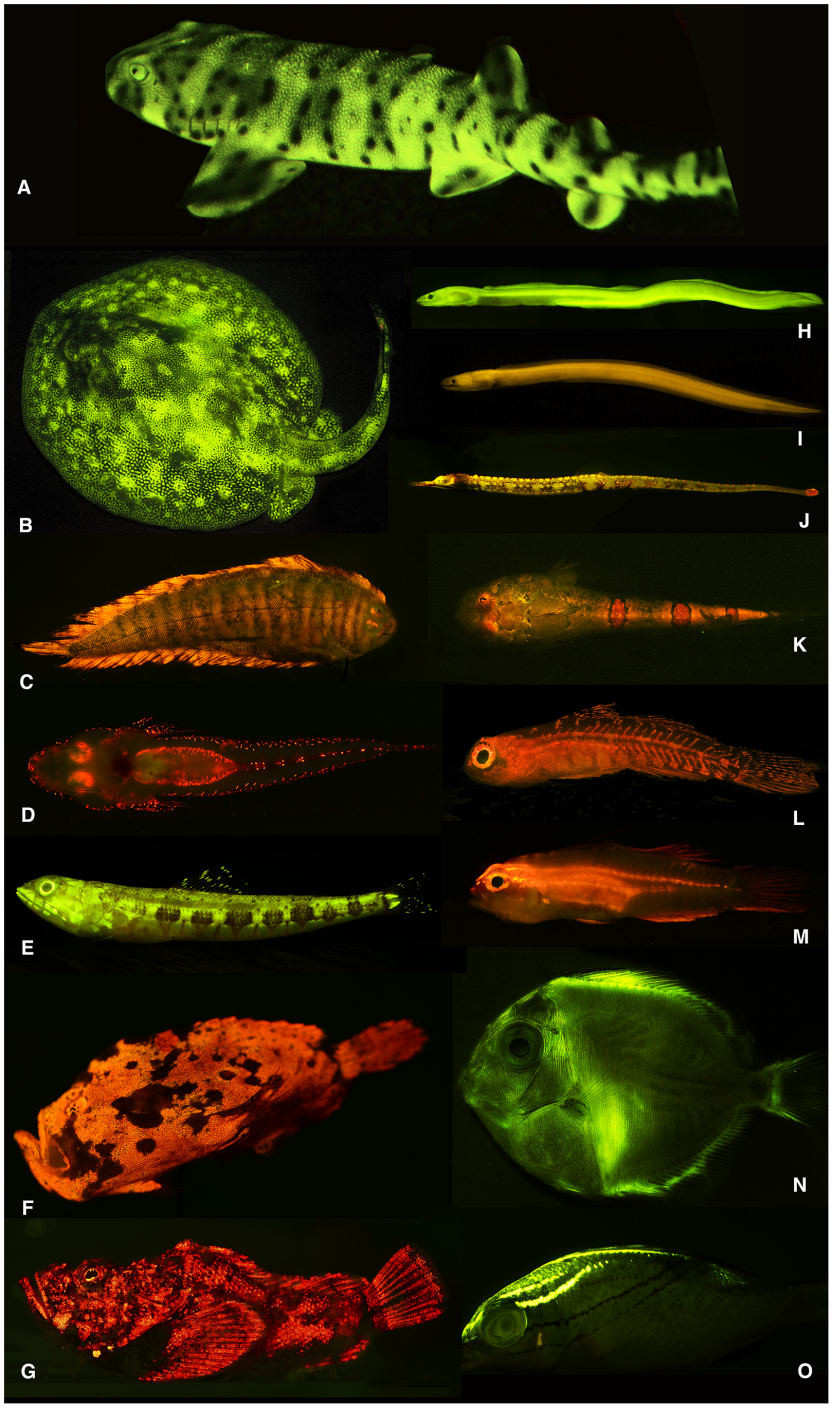
Diversity of fluorescent patterns and colors in marine fishes. A, swell shark (*Cephaloscyllium ventriosum*); B, ray (*Urobatis jamaicensis*); C, sole (*Soleichthys heterorhinos*); D, flathead (*Cociella hutchinsi*); E, lizardfish (*Synodus dermatogenys*); F, frogfish (*Antennarius maculatus*); G, false stonefish (*Scorpaenopsis diabolus*); H, false moray eel (*Kaupichthys brachychirus*); I, false moray eel (*Kaupichthys nuchalis*); J, pipefish (*Corythoichthys haematopterus*); K, sand stargazer (*Gillellus uranidea*); L, goby (*Eviota* sp.); M, goby (*Eviota atriventris*); N, surgeonfish (*Acanthurus coeruleus*, larval); O, threadfin bream (*Scolopsis bilineata*).

All collected specimens were placed on ice to preserve coloration and digitally imaged upon return to shore using Nikon D300s, D7000, or D800 DSLR cameras affixed with either a 60 or 105 mm Nikkor macro lens under white light. Fishes were subsequently scanned for fluorescence using bright LED light sources equipped with excitation filters and observed using emission filter glasses/goggles. All fluorescent fishes were then imaged (Fig. 1) using the “Fluorescent Macro Photography” protocol outlined below.

**Figure 2:**
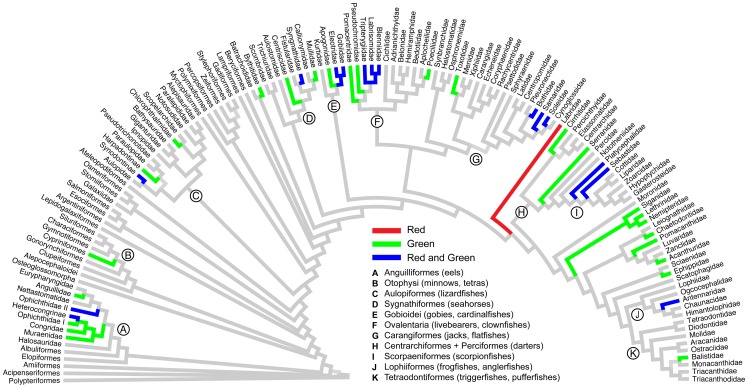
Observed occurrences of green and red fluorescent emissions indicate the evolution of biofluorescence is widespread across the evolutionary history of ray-finned fishes (Actinopterygii). Family-level tree showing evolutionary relationships of ray-finned fishes inferred from maximum likelihood analysis of 221 species and six (one mitochondrial, five nuclear) genes. Note: Not all biofluorescent lineages are shown due to sampling limitations (see Table S1, Fig. S1).

The list and phylogenetic distribution of biofluorescence across cartilaginous and bony fishes presented in Figure 2 and Table S1 are the result of this survey work, and they also include data from [7] that specifically examined red fluorescence in some shallow, reef-associated fishes. In addition, we have summarized other accounts of biofluorescence in fishes from the popular literature (underwater photography magazines and websites) and available on the internet.

### Emission Spectra

Emission spectra were collected using an Ocean Optics USB2000+ miniature spectrometer (Dunedin, FL) equipped with a hand-held fiber optic probe (Ocean Optics ZFQ-12135). Excitation spectra were achieved during illumination with a band-pass filter (450–500 nm, Omega Optical, Inc., Brattleboro, VT, or Semrock, Inc., Rochester, NY). Emission spectra were recorded by applying the fiber optic probe to specific anatomical parts of the individual fish specimen exhibiting biofluorescence. This was repeated several times for each specimen to ensure the accuracy of measurements.

### Fluorescent Macro Photography

Individual fish specimens were placed in a narrow photographic tank and held flat against a thin plate glass front. Fluorescent macro images [7360×4912 (Nikon D800); 4928×3264 (Nikon D7000); 2180×1800 pixel (Nikon D300S)] were produced in a dark room by covering the flash (Nikon SB 600, SB 800, or SB910) with interference bandpass excitation filters (Omega Optical, Inc., Brattleboro, VT; Semrock, Inc., Rochester, NY). Longpass (LP) and bandpass (BP) emission filters (Semrock) were attached to the front of the camera lens. A variety of excitation/emission filter pairs were tested on each sample to elicit the strongest fluorescence emission: excitation 450–500 nm, emission 514 LP; excitation 500–550 nm, emission 561 LP.

### Phylogeny reconstruction

A majority of the DNA sequence data used in this study is from [20], but additional sequences were obtained from many studies [21]–[84]; the GenBank accession numbers for these sequences as well as our added GenBank accession numbers (KF768155-KF768177) can be found in Table S2. Mitochondrial and nuclear genes were aligned using the program MAFFT v6.0 with default parameters [85]. The phylogenetic analysis presented herein had a total of 5,238 base pairs including: one mitochondrial gene (cytochrome oxidase I, 812 bps), and five protein-coding genes (glycosyltransferase gene, 732 bps; myosin heavy chain 6 alpha gene, 737 bps; pleiomorphic adenoma protein-like 2-like gene, 659 bps; recombination activating gene 1, 1403 bps; zic family member protein, 890 bps). For each maximum likelihood analysis, the dataset was partitioned by individual gene fragments. A model of molecular evolution was chosen by the program jMODELTEST v.2.1 [86] with the best fitting model under the Akaike information criteria (AIC) for each individual gene partition assigned, including: cytochrome oxidase I (GTR+I+Γ), glycosyltransferase (GTR+ Γ), myosin heavy chain 6 alpha (GTR+I+Γ), pleiomorphic adenoma protein-like 2-like gene (GTR+I+Γ), recombination activating gene 1 (SYM+I+Γ), and zic family member protein (GTR+I+Γ). Maximum likelihood analyses were performed in GARLI v2.0 [87]. Ten separate analyses were conducted, and the tree having the best likelihood score is presented here (Fig. S1, Fig. 2) to evaluate evolutionary relationships.

## Results

The results presented in this study are based upon ichthyofaunal surveys conducted during multiple expeditions to the Caribbean and tropical Western Pacific (2011–2013), analysis of living aquarium collections, and previous observations of biofluorescence from the literature. Biofluorescence is phylogenetically widespread and phenotypically variable in both cartilaginous (Chondrichthyes: sharks and rays) and bony (ray-finned: e.g, eels, lizardfishes, gobies, flatfishes) fishes (Figs. 1, 2, Table S1). We find biofluorescence to be most common and morphologically variable in cryptically pigmented and patterned marine lineages, including true eels (Anguilliformes), lizardfishes (Aulopiformes), scorpionfishes (Scorpaenoidei), blennies (Blennioidei), gobies (Gobioidei), and flatfishes (Pleuronectiformes) (Figs. 1, 2), groups that generally appear well camouflaged in the reef environment. With our initial surveys, we have already identified 16 orders, 50 families, 105 genera, and more than 180 species of biofluorescent fishes, and we have reconstructed our current understanding of the phylogenetic distribution of biofluorescence for ray-finned fishes (Fig. 2, Table S1).

**Figure 3:**
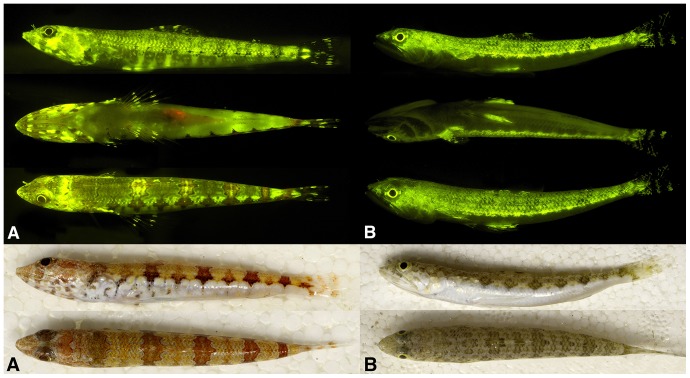
Top panel: Interspecific variation in fluorescent emission pattern (from top: lateral, ventral, and dorsal views) in two congeneric and sympatric members of the lizardfish genus *Synodus*. A, *S. synodus*. B, *S. saurus*. Bottom panel: Interspecific variation in coloration and pigmentation pattern under white light (top: lateral; bottom: dorsal) in same two congeneric and sympatric members of the lizardfish genus *Synodus*. A, *S. synodus*. B, *S. saurus*.

We show that besides red fluorescence previously reported in shallow reef-associated fishes (e.g., [7], [88]), marine fishes also commonly exhibit green fluorescence, or combinations of green and red or orange fluorescence in unique, species-specific patterns (Figs. 1, 3). Biofluorescent patterning in fishes ranges from simple red, orange or green eye rings to striking, complex, species-specific patterns of interspersed fluorescent elements, frequently comprising multiple colors, on the head, jaws, fins, flank, and ventrum—and even bright fluorescence of the entire body (e.g., chlopsid eels; Fig. 1). Considerable interspecific variation in fluorescent emission patterns are recorded for members of the lizardfish genus *Synodus* (Fig. 3) and the goby genus *Eviota* (Fig. 1L, M), even among closely related species that appear nearly identical under white light (Fig. 3A, B).

## Discussion

We find biofluorescence to be widespread across cartilaginous and bony fishes, and we show that this evolutionary phenomenon is most common and phenotypically variable in cryptically colored and patterned marine fishes, such as eels, lizardfishes, blennies, scorpionfishes, gobies, and flatfishes (Figs. 1, 2). The repeated evolution of biofluorescence combined with phenotypically variable coloration (green, orange, red) and patterns in fishes may suggest a previously unrecognized role in communication, including mating behavior as has been observed in parrots [9]. Fluorescence may be exploited in fishes to produce visual contrast and patterns in otherwise cryptically patterned or camouflaged species that blend in well on the reef in shallow sunlit waters.

A few instances of green biofluorescence have also been reported in deepwater (500–600 m) catsharks (Scyliorhinidae), lizardfishes (Aulopiformes: Chlorophthalmidae), and an unidentified ceriantharian (Cnidaria) [88]–[89]. The presence of biofluorescence in these deepwater taxa that spend their lives primarily in the dark, beyond the reach of the high-energy blue light necessary for excitation of fluorescence, is curious from a functional perspective. Biofluorescence in these taxa potentially represents the ancestral condition in lineages whose shallower water relatives also exhibit biofluorescence (Figs. 1, 2). Some bioluminescent (production and emission of light through a chemical reaction) deep-sea organisms have previously been shown to exhibit biofluorescence through a coupling of both bioluminescent and biofluorescent systems. A heavily studied example is the crystal jellyfish (*Aequorea victoria*) in which the bioluminescent system (aequorin) produces blue light that directly excites green fluorescent protein (GFP) to emit green light [90], likely via a Förster energy transfer process [91]. In another example, the deep-sea loose-jaw dragonfish (*Malacosteus*) emits red light through biofluorescence via the absorption of blue bioluminescent light produced by the fish, which is reemitted by a chlorophyll-like compound as red light and is hypothesized to aid in predation [92]. In addition, some deep-sea siphonophores also utilize bioluminescent light to excite red biofluorescence [12].

Shallow water bony fishes generally exhibit good color vision [93]–[94], a result of living in a visually complex environment; in contrast, fishes occurring in deeper water exhibit limited color vision due to a simpler (blue-shifted) visual environment. Recent evidence indicates that sharks and rays also exhibit color vision [95]–[96]. Many of the fishes we find to exhibit biofluorescence (Figs. 1, 2), such as sharks, lizardfishes, scorpionfishes, labrids (wrasses), and flatfishes, also possess yellow intraocular filters [18]. Yellow intraocular filters in the lenses and corneas of certain fishes function as long-pass filters, thus enabling the species that possess them to visualize and potentially exploit fluorescence to enhance visual contrast and patterns that are unseen to other fishes and predators that lack this visual specialization.

It has been hypothesized that some polarization sensitive cephalopods communicate via “private” polarized light signals that allow them to simultaneously remain camouflaged to predators [97] and exploit a “hidden” communication mechanism between conspecifics [98]. Cephalopods possess a rhabdomeric visual system that enables detection of linearly polarized light and they are able to produce polarized skin patterns using iridophores [99], whereas many of their predators (marine mammals and some fishes) are not sensitive to the polarization of light [100]. Likewise, fishes that possess the necessary yellow intraocular filters for visualizing biofluorescence could be exploiting a similar “hidden” light signal for a similar functional role. We found that biofluorescent patterning was especially prominent in cryptically patterned fishes, and that many of these lineages also possess yellow long-pass intraocular filters that could enable visualization of such patterns (Figs. 1, 2).

In recent years, biofluorescence has also been found in patchy occurrences in some copepods [4] and mantis shrimp (phylum Arthropoda) [5], amphioxus (phylum Chordata) [6], and a species of comb jelly (phylum Ctenophora) [3]. Biofluorescence has been shown to enhance signaling in the mantis shrimp, *Lysiosquillina glabriuscula*, a species identified to have a complex system of color visualization [5]. Additionally, there have been reports of fluorescence signaling in butterflies [8], parrots [9], spiders [10], and flowers [11], as well as in a deep-sea siphonophore [12].

**Figure 4:**
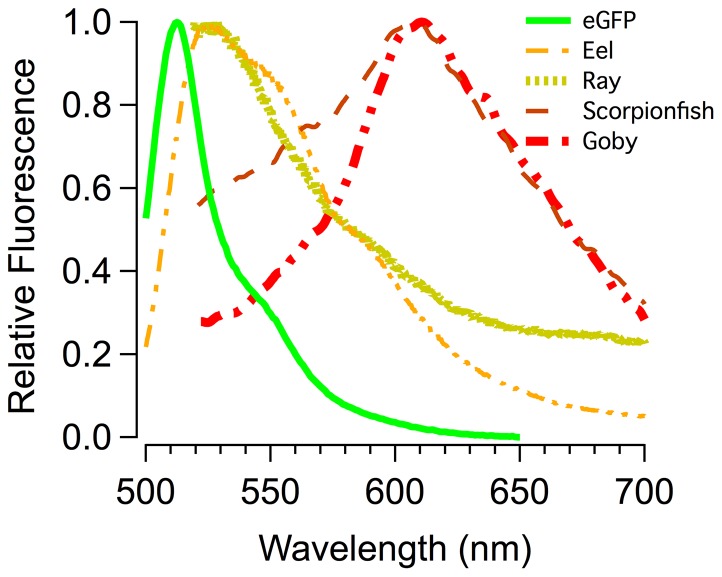
Plot of emission spectra for representative green and red fluorescing marine fishes, also showing the spectra for enhanced green fluorescent protein (eGFP) for comparison. Key to species sampled: Ray (family Urotrygonidae, genus *Urobatis*); Eel (family Chlopsidae, genus *Kaupichthys*); Scorpionfish (family Scorpaenidae, genus *Scorpeana*); Goby (family Gobiidae, genus *Eviota*).

The phylogeny presented in Figure 1 indicates that biofluorescence is phylogenetically widespread and phenotypically variable across ray-finned fishes (Actinopterygii) in terms of the diversity of patterns observed (Figs. 1, 3), emission spectra (Fig. 4), and intensity. We observed distinct variation among lineages and pronounced interspecific variation in emission patterns in closely related taxa that otherwise look nearly identical under white light. For example, closely related lizardfish species within the genus *Synodus* exhibit fluorescence patterns that are notably more distinct than their pigmentation patterns appear under daylight/white light (Fig. 3). Considerable interspecific fluorescent pattern variation is also observed across species in the goby genus *Eviota* (Fig. 1L, M) and for chlopsid eels (Anguilliformes: Chlopsidae; Fig. 1H, I). Our observations indicate that flatfishes exhibit distinctly different fluorescent patterns on their sighted and blind surfaces (fluorescence on the sighted side being primarily red (Fig. 1B), whereas the blind side generally fluoresces green), which is intriguing given that flatfishes are well known to flash their blind sides to each other during mating rituals. Individuals of some other species were found to exhibit both alternating red and green fluorescent patterns (e.g., Fig. 1K), whereas in other lineages, only the larval forms were observed to fluoresce (e.g., Fig. 1N, acanthurids). Such observations in combination with pronounced interspecific variability in fluorescence emission pattern in otherwise similarly patterned taxa suggest that intraspecific communication is a function of biofluorescence in marine fishes, as has been shown in other organisms with complex visual systems (e.g., [9]). In addition, certain marine fishes (e.g., [101], [102]) spawn synchronously surrounding the full moon. Moonlight illumination in shallow ocean waters could potentially provide the appropriate excitation energy for green and red biofluorescence in fishes, and as a result, species-specific biofluorescent patterning may provide an added layer of species recognition during the spawning phase, when fishes are particularly vulnerable to predation.

**Figure 5:**
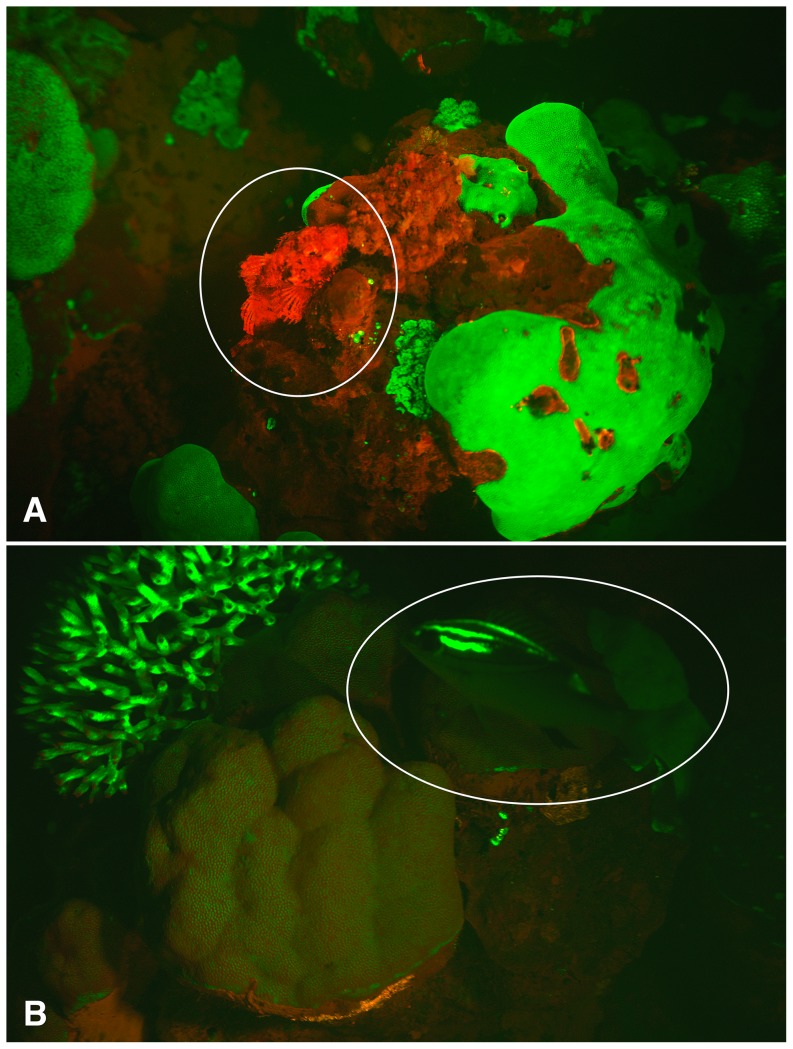
Images of reef fishes fluorescing in their natural habitat captured with a Red Epic video camera at night in the Solomon Islands. (A) A red fluorescing scorpionfish, *Scorpaenopsis papuensis*, perched on red fluorescing algae. (B) A green fluorescing nemipterid (bream), *Scolopsis bilineata*, near a green fluorescing *Acropora* sp. coralhead.

In addition, we present evidence that some fish lineages might be utilizing fluorescence as a means of camouflage in specific marine environments (Fig. 5; Videos S1, S2). Red and far-red biofluorescence is a ubiquitous feature of photosynthetic organisms due to the properties of chlorophyll and other photosynthetic pigment complexes. The photosynthetic apparatus associated with chlorophyll fluoresces red and provides a background of biofluorescence in areas of algal growth. Apart from photosynthetic organisms, red biofluorescence also occurs due to fluorescent proteins [4]. In two species of red biofluorescent scorpionfishes that we imaged, individuals were observed residing on top of a patch of red fluorescing algae (Fig. 5A). We also recorded a bream (*Scolopsis*) with green fluorescent patterns on its nape swimming within a green fluorescing *Acropora* coral outcrop (Fig. 5B). It would appear that under fluorescent conditions, these species are particularly well camouflaged in the specific environments in which they were imaged.

In summary, the widespread nature of biofluorescence in both cartilaginous and bony, ray-finned marine fishes, coupled with the presence of yellow intraocular filters in many biofluorescent lineages that would permit the visualization of fluorescent emissions, is intriguing. Biofluorescence is most prominent and phenotypically variable in cryptically patterned, well-camouflaged lineages (Figs. 1, 2) that otherwise blend in with their surroundings. Coupled with observations of notably distinct fluorescent emission patterns among closely related species (including sister species) that otherwise strongly resemble each other under white light/daylight (Figs. 2, 3), suggests a intraspecific communication/species recognition function. Conversely, we observed species that appear to blend in with their surroundings under fluorescent lighting conditions (Fig. 5), and that could theoretically exploit biofluorescence as a means of camouflage to either avoid being detected by potential prey or to elude predators. Based on these data, the possibility exists that marine fishes are using biofluorescence for a variety of functions, including communication (species recognition, mating), predator avoidance, and potentially even prey attraction/predation. The broad phylogenetic distribution of biofluorescence across bony fishes is consistent with its repeated independent evolution, and its importance in the diversification of marine fishes remains to be explored. As Johnsen [103] justly notes, the field of biofluorescence is wide open for study and there have been far too few studies to date, most of which have focused on cnidarians. With the recent discovery of a novel fluorescent protein from a vertebrate [14], we expect that biofluorescence in marine fishes will be the subjects of many future studies, from the level of proteins to whole organisms in their environment.

## Supporting Information

Figure S1 Maximum likelihood topology of the evolutionary relationships of ray-finned fishes inferred from the analysis of 221 species (representing more than 145 families), with six gene fragments (one mitochondrial, five nuclear).
(PDF)

Table S1 Biofluorescent fishes known to date. Taxa are listed alphabetically by Order (column 1), Family (column 2), and Species (column 3). Columns 4 (red) and 5 (green) contain filled circles corresponding to the observed color of fluoresced light. Column 6 gives AMNH catalog numbers. Taxa indicated with an * are not included in the phylogenetic reconstruction (Fig. 2).
(DOCX)

Table S2 GenBank accession numbers and sources for DNA sequences utilized in the phylogenetic reconstruction shown in Fig. 2 and Fig. S1.
(PDF)

Video S1 Supplementary video to accompany Fig. 5A showing a red fluorescing scorpionfish, *Scorpaenopsis papuensis*, perched on red fluorescing algae in its natural habitat. Video captured with a Red Epic video camera at night in the Solomon Islands.
(MOV)

Video S2 Supplementary video to accompany Fig. 5B showing a green fluorescing nemipterid (bream), *Scolopsis bilineata*, swimming near a green fluorescing *Acropora sp.* coralhead. Video captured with a Red Epic video camera at night in the Solomon Islands.
(MOV)
