# Supplementary figures and images for: The Covert World of Fish Biofluorescence: A Phylogenetically Widespread and Phenotypically Variable Phenomenon
Source: PLoS One. 2014 Jan 8;9(1):e83259. doi: 10.1371/journal.pone.0083259 (PMC3885428; doi:10.1371/journal.pone.0083259)

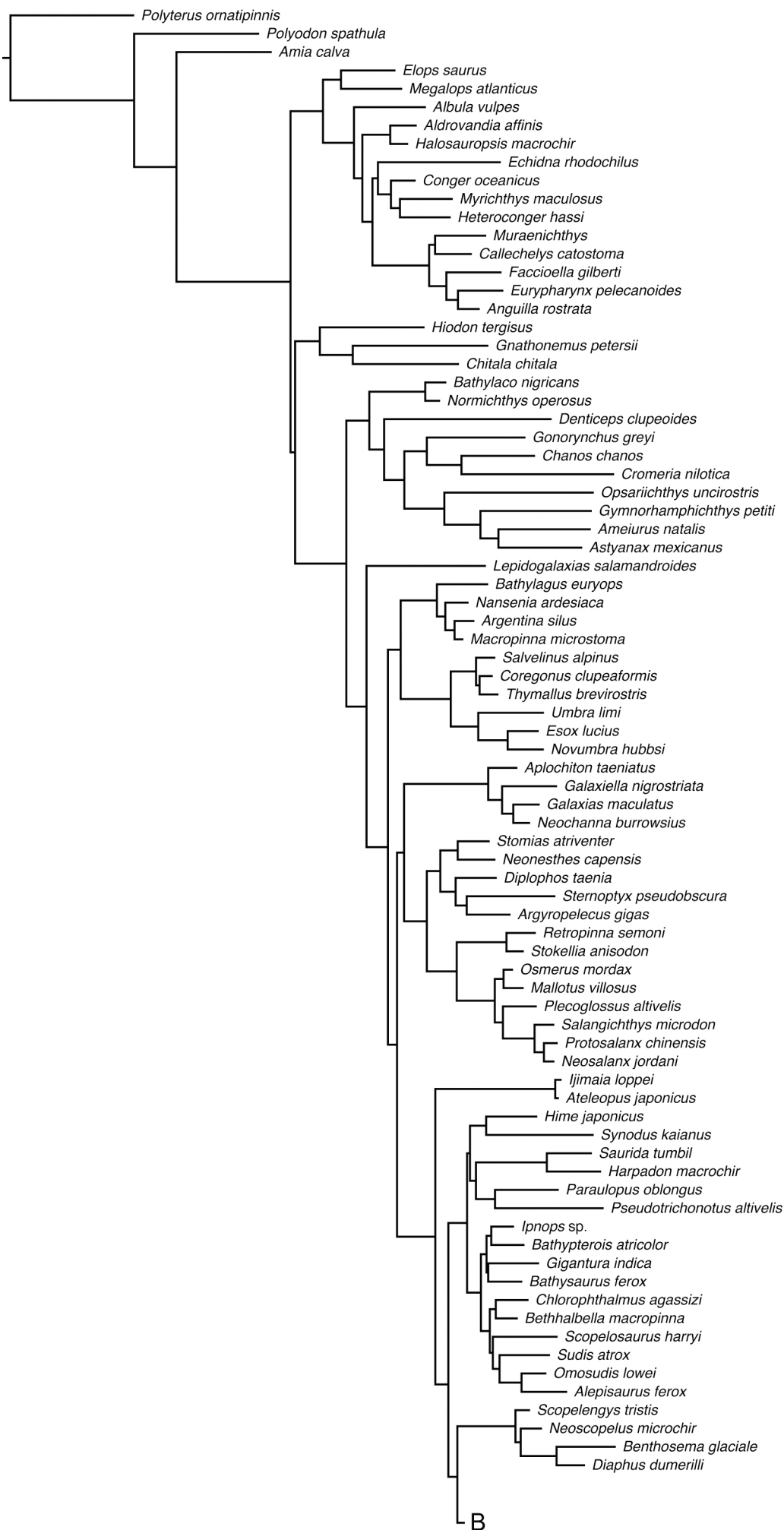

Supplementary Figure 1

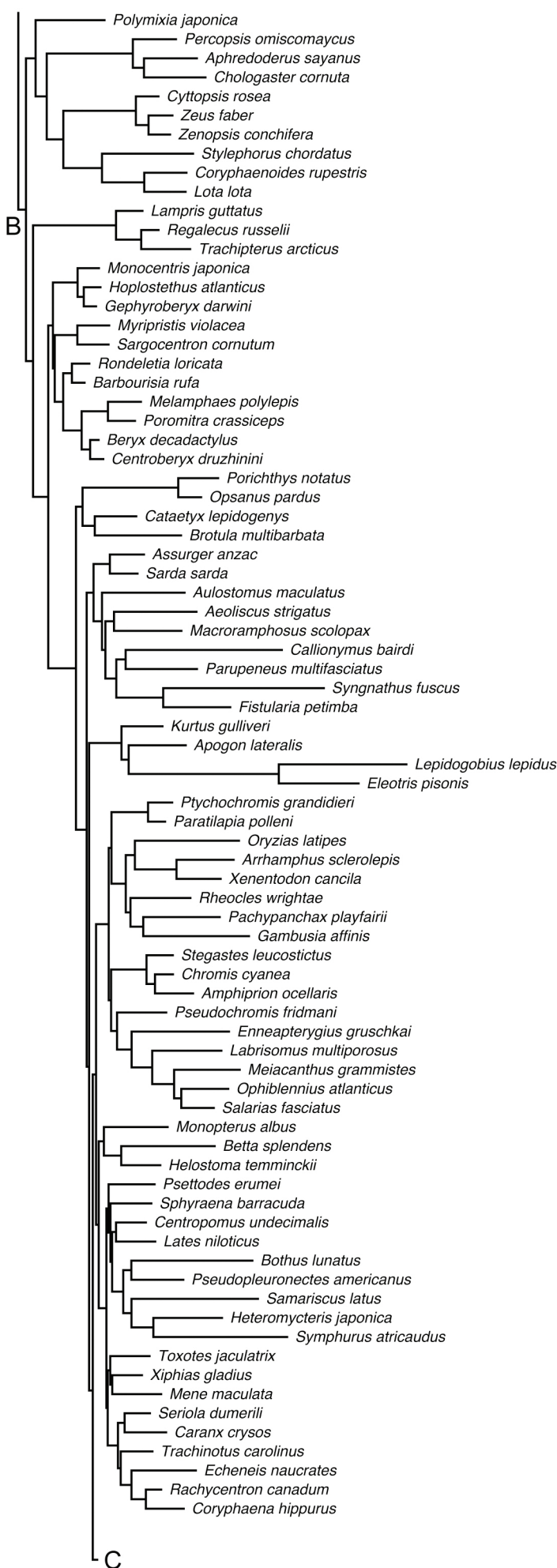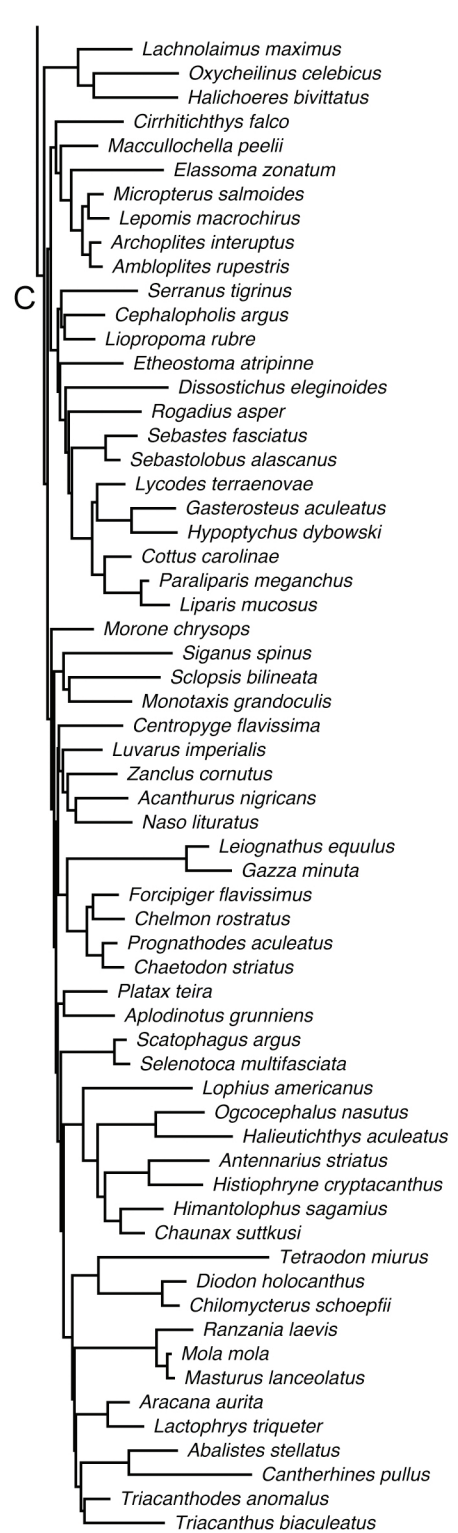

Supplement: Figure S1 — Maximum likelihood topology of the evolutionary relationships of ray-finned fishes inferred from the analysis of 221 species (representing more than 145 families), with six gene fragments (one mitochondrial, five nuclear). (PDF) [file pone.0083259.s001.pdf]
