# Supplementary material for: The Covert World of Fish Biofluorescence: A Phylogenetically Widespread and Phenotypically Variable Phenomenon
Source: PLoS One. 2014 Jan 8;9(1):e83259. doi: 10.1371/journal.pone.0083259 (PMC3885428; doi:10.1371/journal.pone.0083259)
Supplement: Table S1 — Biofluorescent fishes known to date. Taxa are listed alphabetically by Order (column 1), Family (column 2), and Species (column 3). Columns 4 (red) and 5 (green) contain filled circles corresponding to the observed color of fluoresced light. Column 6 gives AMNH catalog numbers. Taxa indicated with an * are not included in the phylogenetic reconstruction (Fig. 2). (DOCX) [file pone.0083259.s002.docx]

**Supplementary Table 1.** Biofluorescent fishes. Taxa are listed alphabetically by Order (column 1), Family (column 2), and Species (column 3). Columns 4 (red) and 5 (green) contain filled circles corresponding to the observed color of fluoresced light. Column 6 gives AMNH catalog numbers. Taxa indicated with an * are not included in the phylogenetic reconstruction (Fig. 2).

**Chondrichthyes, Elasmobranchii:**

| Carchariniformes | Scyliorhinidae | *Cephaloscyllium ventriosum* * |  | **•** |  |
| --- | --- | --- | --- | --- | --- |
| Carchariniformes | Scyliorhinidae | *Scyliorhinus retifer* * |  | **•** |  |
| Myliobatiformes | Urotrygonidae | *Urobatis jamaicensis* * |  | **•** |  |

**Osteichthyes:**

| Anguilliformes | Anguillidae | *Anguilla* sp. |  | **•** |  |
| --- | --- | --- | --- | --- | --- |
| Anguilliformes | Chlopsidae | *Kaupichthys brachychirus* * | **•** | **•** | 259838 |
| Anguilliformes | Chlopsidae | *Kaupichthys hyoproroides* * | **•** | **•** |  |
| Anguilliformes | Chlopsidae | *Kaupichthys diodontus* * |  | **•** | 256030, 259870 |
| Anguilliformes | Chlopsidae | *Kaupichthys nuchalis* * | **•** | **•** | 252011 |
| Anguilliformes | Congridae | *Conger cinereus* |  | **•** | 256081 |
| Anguilliformes | Congridae | *Conger triporiceps* |  | **•** | 252010 |
| Anguilliformes | Congridae | *Heteroconger hassi* |  | **•** |  |
| Anguilliformes | Muraenidae | *Gymnomuraena zebra* |  | **•** | 256035 |
| Anguilliformes | Muraenidae | *Gymnothorax pictus* |  | **•** | 256023 |
| Anguilliformes | Muraenidae | *Gymnothorax robinsi* |  | **•** | 259861 |
| Anguilliformes | Muraenidae | *Gymnothorax* sp. |  | **•** | 259863 |
| Anguilliformes | Muraenidae | *Muraena lentiginosa* |  | **•** |  |
| Anguilliformes | Muraenidae | *Uropterygius concolor* |  | **•** | 256066 |
| Anguilliformes | Ophichthidae | *Ahlia egmontis* | **•** | **•** |  |
| Anguilliformes | Ophichthidae | *Muraenichthys schultzei* | **•** | **•** | 259839, 259840 |
| Anguilliformes | Ophichthidae | *Myrichthys breviceps* |  | **•** |  |
| Anguilliformes | Ophichthidae | *Myrichthys colubrinus* |  | **•** | 259849 |
| Anguilliformes | | Glass eel (juvenile) |  | **•** | 259857 |
| Atheriniformes | Melanotaeniidae | *Melanotaenia boesemani* * |  | **•** |  |
| Aulopiformes | Chlorophthalmidae | *Chlorophthalmus* sp. |  | **•** |  |
| Aulopiformes | Synodontidae | *Saurida gracilis* |  | **•** | 256048, 256055, 256056, 259830, 259847 |
| Aulopiformes | Synodontidae | *Synodus dermatogenys* | **•** | **•** | 256149, 259866, 259869 |
| Aulopiformes | Synodontidae | *Synodus lucioceps* |  | **•** |  |
| Aulopiformes | Synodontidae | *Synodus rubromarmoratus* |  | **•** | 259826, 259851 |
| Aulopiformes | Synodontidae | *Synodus saurus* | **•** | **•** |  |
| Aulopiformes | Synodontidae | *Synodus synodus* | **•** | **•** |  |
| Aulopiformes | Synodontidae | *Synodus variegatus* |  | **•** | 256065 |
| Cyprinodontiformes | Poeciliidae | *Xiphophorus* cf. *hellerii* |  | **•** |  |
| Cypriniformes | Cyprinidae | *Tanichthys albonubes* |  | **•** |  |
| Gasterosteiformes | Pegasidae | *Eurypegasus draconis* * | **•** |  |  |
| Gobiesociformes | Gobiesocidae | *Arcos* sp. * |  | **•** |  |
| Gobiesociformes | Gobiesocidae | *Gobiesox* sp. * |  | **•** |  |
| Lophiiformes | Antennariidae | *Antennarius maculatus* | **•** |  | 259858 |
| Lophiiformes | Antennariidae | *Antennarius randalli* |  | **•** | 259859 |
| Ophidiiformes | Carapidae | Juvenile carapid * |  | **•** | 259874 |
| Ophidiiformes | Bythitidae | Bythitid sp. |  | **•** | 259836 |
| Ophidiiformes | Bythitidae | Bythitid sp. |  | **•** | 259841 |
| Perciformes | Acanthuridae | *Acanthurus coeruleus* |  | **•** |  |
| Perciformes | Acanthuridae | *Acanthurus pyroferus* |  | **•** | 256029 |
| Perciformes | Apogonidae | *Pristiapogon abrogramma* |  | **•** | 259817 |
| Perciformes | Apogonidae | Apogonid sp. |  | **•** | 259845 |
| Perciformes | Blenniidae | *Crossosalarias macrospilus* | **•** |  |  |
| Perciformes | Blenniidae | *Ecsenius dentex* | **•** |  |  |
| Perciformes | Blenniidae | *Ecsenius pictus* |  | **•** | 256064, 259848 |
| Perciformes | Blenniidae | *Salarias segmentatus* |  | **•** | 259873 |
| Perciformes | Chaetodontidae | *Chaetodon citrinellus* |  | **•** | 256078 |
| Perciformes | Chaetodontidae | *Chaetodon octofasciatus* |  | **•** | 256059 |
| Perciformes | Chaetodontidae | *Forcipiger longirostris* |  | **•** | 256032 |
| Perciformes | Chaetodontidae | *Heniochus varius* | **•** | **•** | 256036 |
| Perciformes | Cirrhitidae | *Cirrhitichthys falco* | **•** |  | 256039 |
| Perciformes | Creediidae | *Limnichthys nitidus* * | **•** |  |  |
| Perciformes | Dactyloscopidae | *Gillellus uranidea* * | **•** |  |  |
| Perciformes | Ephippidae | *Platax boersii* |  | **•** | 256037 |
| Perciformes | Gobiidae | *Bryaninops natans* | **•** |  |  |
| Perciformes | Gobiidae | *Bryaninops ridens* | **•** |  |  |
| Perciformes | Gobiidae | *Bryaninops yongei* | **•** |  |  |
| Perciformes | Gobiidae | *Callogobius stellatus* |  | **•** | 259843 |
| Perciformes | Gobiidae | *Coryphopterus* | **•** | **•** | 256147 |
| Perciformes | Gobiidae | *Ctenogobiops maculosus* | **•** |  |  |
| Perciformes | Gobiidae | *Ctenogobiops mitodes* | **•** | **•** | 259837 |
| Perciformes | Gobiidae | *Ctenogobiops tangaroai* | **•** |  |  |
| Perciformes | Gobiidae | *Eviota atriventris* | **•** |  | 256145, 259803, 259804, 259820, 259822, 259853 |
| Perciformes | Gobiidae | *Eviota bifasciata* | **•** |  | 259833 |
| Perciformes | Gobiidae | *Eviota distigma* | **•** |  |  |
| Perciformes | Gobiidae | *Eviota dorsogilva* | **•** | **•** | 259814, 259842 |
| Perciformes | Gobiidae | *Eviota guttata* | **•** |  |  |
| Perciformes | Gobiidae | *Eviota melasma* | **•** |  | 259806, 259821, 259825 |
| Perciformes | Gobiidae | *Eviota nigriventris* | **•** |  |  |
| Perciformes | Gobiidae | *Eviota pellucida* | **•** |  |  |
| Perciformes | Gobiidae | *Eviota prasina* | **•** |  |  |
| Perciformes | Gobiidae | *Eviota queenslandica* | **•** |  |  |
| Perciformes | Gobiidae | *Eviota sebreei* | **•** |  |  |
| Perciformes | Gobiidae | *Eviota sparsa* | **•** | **•** | 256144, 259805, 259831, 259865 |
| Perciformes | Gobiidae | *Eviota zebrina* | **•** |  |  |
| Perciformes | Gobiidae | *Eviota* sp. | **•** |  | 259807 |
| Perciformes | Gobiidae | *Eviota* sp. | **•** | **•** | 259832 |
| Perciformes | Gobiidae | *Eviota* sp. | **•** |  | 259860 |
| Perciformes | Gobiidae | *Eviota* sp. | **•** |  | 259867 |
| Perciformes | Gobiidae | *Fusigobius duospilus* | **•** |  |  |
| Perciformes | Gobiidae | *Fusigobius longispinus* | **•** |  |  |
| Perciformes | Gobiidae | *Fusigobius neophytus* | **•** | **•** | 259835 |
| Perciformes | Gobiidae | *Gladiogobius ensifer* | **•** |  | 256060 |
| Perciformes | Gobiidae | *Gnatholepis anjerensis* | **•** |  |  |
| Perciformes | Gobiidae | *Gobiodon ceramensis* |  | **•** | 259872 |
| Perciformes | Gobiidae | *Gobiodon citrinus* | **•** | **•** | 259801 |
| Perciformes | Gobiidae | *Gobiodon rivulatus* | **•** | **•** | 259852 |
| Perciformes | Gobiidae | *Istigobius decoratus* | **•** |  |  |
| Perciformes | Gobiidae | *Paragobiodon xanthosoma* |  | **•** | 259844 |
| Perciformes | Gobiidae | *Pleurosicya micheli* | **•** | **•** | 259808, 259829 |
| Perciformes | Gobiidae | *Pleurosicya prognatha* | **•** |  |  |
| Perciformes | Gobiidae | *Pleurosicya* sp. | **•** |  | 259816 |
| Perciformes | Gobiidae | *Trimma avidori* | **•** |  |  |
| Perciformes | Gobiidae | *Trimma benjamini* |  | **•** | 259823, 259824 |
| Perciformes | Gobiidae | *Trimma macrophthalmum* |  | **•** | 259811 |
| Perciformes | Gobiidae | *Trimma milta* |  | **•** | 259810 |
| Perciformes | Gobiidae | *Trimma striata* |  | **•** | 259834 |
| Perciformes | Gobiidae | *Trimma* sp. |  | **•** | 259802 |
| Perciformes | Gobiidae | *Trimma* sp. |  | **•** | 259812 |
| Perciformes | Gobiidae | *Trimma* sp. |  | **•** | 259846 |
| Perciformes | Gobiidae | *Trimma* sp. |  | **•** | 259864 |
| Perciformes | Gobiidae | Gobiid sp. | **•** |  | 259809 |
| Perciformes | Gobiidae | Gobiid sp. |  | **•** | 259827 |
| Perciformes | Labridae | *Cheilinus fasciatus* |  | **•** | 256082 |
| Perciformes | Labridae | *Labroides dimidiatus* | **•** |  | 256043, 256075 |
| Perciformes | Labridae | *Oxycheilinus celebicus* |  | **•** | 256040 |
| Perciformes | Labridae | *Paracheilinus octotaenia* | **•** |  |  |
| Perciformes | Labridae | *Pseudocheilinus evanidus* | **•** | **•** | 256062, 256068, 256083 |
| Perciformes | Labridae | *Semicossyphus pulcher* |  | **•** |  |
| Perciformes | Labrisomidae | *Malacoctenus* sp. | **•** |  |  |
| Perciformes | Labrisomidae | *Malacoctenus versicolor* | **•** |  | 255805, 255812, 255901, 255925, 256016 |
| Perciformes | Labrisomidae | *Paraclinus marmoratus* | **•** |  |  |
| Perciformes | Labrisomidae | *Paraclinus nigripinnis* |  | **•** |  |
| Perciformes | Lethrinidae | *Monotaxis grandoculis* |  | **•** | 256073 |
| Perciformes | Microdesmidae | *Nemateleotris magnifica* * |  | **•** | 259854 |
| Perciformes | Mullidae | *Parupeneus multifasciatus* |  | **•** | 256049 |
| Perciformes | Mullidae | *Pseudupeneus maculatus* |  | **•** |  |
| Perciformes | Nemipteridae | *Pentapodus trivittatus* |  | **•** | 256052, 256053 |
| Perciformes | Nemipteridae | *Scolopsis bilineata* |  | **•** | 256054 |
| Perciformes | Nemipteridae | *Scolopsis ciliata* |  | **•** | 256072 |
| Perciformes | Nemipteridae | *Scolopsis lineata* |  | **•** |  |
| Perciformes | Nemipteridae | *Scolopsis margaritifer* |  | **•** | 256050 |
| Perciformes | Pomacanthidae | *Centropyge bicolor* |  | **•** | 256069 |
| Perciformes | Pomacanthidae | *Centropyge vrolikii* |  | **•** | 256076 |
| Perciformes | Pomacentridae | *Amphiprion clarkii* |  | **•** | 256044 |
| Perciformes | Pomacentridae | *Amphiprion percula* |  | **•** | 256038, 256042 |
| Perciformes | Pseudochromidae | *Pseudochromis bitaeniatus* |  | **•** | 259813 |
| Perciformes | Sparidae | *Sparisoma radians* * |  | **•** |  |
| Perciformes | Serranidae | *Cephalopholis leopardus* |  | **•** | 256031 |
| Perciformes | Serranidae | *Liopropoma multilineatum* |  | **•** | 256063 |
| Perciformes | Serranidae | *Plectranthias inermis* | **•** |  |  |
| Perciformes | Serranidae | *Plectranthias nanus* |  | **•** | 256146 |
| Perciformes | Serranidae | *Pseudanthias tuka* |  | **•** | 256033, 256034 |
| Perciformes | Serranidae | *Pseudogramma polyacantha* |  | **•** | 256061 |
| Perciformes | Serranidae | *Pseudogramma* sp. |  | **•** | 259871 |
| Perciformes | Serranidae | *Rypticus subbifrenatus* |  | **•** | 255757, 255847 |
| Perciformes | Serranidae | *Serranus tigrinus* |  | **•** | 255779, 255908 |
| Perciformes | Siganidae | *Siganus canaliculatus* |  | **•** | 256026 |
| Perciformes | Tripterygiidae | *Enneanectes atrorus* | **•** | **•** | 255844 |
| Perciformes | Tripterygiidae | *Enneapterygius abeli* | **•** |  |  |
| Perciformes | Tripterygiidae | *Enneapterygius destai* | **•** |  |  |
| Perciformes | Tripterygiidae | *Enneapterygius flavoccipitis* | **•** |  | 259868 |
| Perciformes | Tripterygiidae | *Enneapterygius mirabilis* | **•** |  |  |
| Perciformes | Tripterygiidae | *Enneapterygius pusillus* | **•** |  |  |
| Perciformes | Tripterygiidae | *Helcogramma novaecaledoniae* | **•** |  | 259862 |
| Perciformes | Tripterygiidae | *Helcogramma steinitzi* | **•** |  |  |
| Perciformes | Tripterygiidae | *Ucla xenogrammus* | **•** |  | 259828, 259855 |
| Pleuronectiformes | Bothidae | *Asterorhombus cocosensis* | **•** | **•** |  |
| Pleuronectiformes | Samaridae | *Samariscus triocellatus* | **•** | **•** | 256047 |
| Pleuronectiformes | Soleidae | *Soleichthys heterorhinos* | **•** | **•** | 256045, 259850 |
| Pleuronectiformes | Soleidae | *Soleichthys oculofasciatus* | **•** |  | 256150 |
| Scorpaeniformes | Hemitripteridae | *Nautichthys oculofasciatus* * |  | **•** |  |
| Scorpaeniformes | Hexagrammidae | *Oxylebius pictus* * | **•** |  |  |
| Scorpaeniformes | Platycephalidae | *Cociella hutchinsi* | **•** | **•** | 256051 |
| Scorpaeniformes | Platycephalidae | *Cymbacephalus beauforti* | **•** | **•** | 256046, 256148 |
| Scorpaeniformes | Platycephalidae | Platycephalid sp. (juvenile) | **•** |  | 259815 |
| Scorpaeniformes | Platycephalidae | Platycephalid sp. (juvenile) | **•** | **•** | 259856 |
| Scorpaeniformes | Scorpaenidae | *Pterois antennata* * | **•** |  | 256041 |
| Scorpaeniformes | Scorpaenidae | *Scorpaena guttata* * | **•** |  |  |
| Scorpaeniformes | Scorpaenidae | *Scorpaena plumieri* * | **•** |  |  |
| Scorpaeniformes | Scorpaenidae | *Scorpaenodes caribbaeus* * | **•** |  |  |
| Scorpaeniformes | Scorpaenidae | *Scorpaenodes hirsutus* * |  | **•** | 256057 |
| Scorpaeniformes | Scorpaenidae | *Scorpaenodes varipinnis* * |  | **•** | 256058 |
| Scorpaeniformes | Scorpaenidae | *Scorpaenopsis diabolus* * | **•** |  |  |
| Scorpaeniformes | Scorpaenidae | *Scorpaenopsis papuensis* * | **•** |  | 256067, 256080 |
| Scorpaeniformes | Scorpaenidae | *Sebastapistes strongia* * | **•** | **•** | 256074 |
| Scorpaeniformes | Sebastidae | *Sebastes dallii* |  | **•** |  |
| Scorpaeniformes | Sebastidae | *Sebastes constellatus* | **•** |  |  |
| Scorpaeniformes | Sebastidae | *Sebastes maliger* |  | **•** |  |
| Scorpaeniformes | Sebastidae | *Sebastes pinniger* | **•** |  |  |
| Syngnathiformes | Centriscidae | *Aeoliscus strigatus* |  | **•** | 256022 |
| Syngnathiformes | Syngnathidae | *Corythoichthys flavofasciatus* | **•** |  |  |
| Syngnathiformes | Syngnathidae | *Corythoichthys haematopterus* | **•** |  | 256070, 256071 |
| Syngnathiformes | Syngnathidae | *Corythoichthys schultzi* | **•** |  |  |
| Syngnathiformes | Syngnathidae | *Doryrhamphus melanopleura* | **•** |  |  |
| Syngnathiformes | Syngnathidae | *Dunkerocampus pessuliferus* | **•** |  |  |
| Syngnathiformes | Syngnathidae | *Hippocampus erectus* | **•** |  |  |
| Syngnathiformes | Syngnathidae | *Hippocampus zosterae* |  | **•** |  |
| Syngnathiformes | Syngnathidae | *Syngnathoides biaculeatus* | **•** |  | 256079 |
| Tetraodontiformes | Balistidae | *Balistapus undulatus* |  | **•** | 256025 |
