# Supplementary material for: The Covert World of Fish Biofluorescence: A Phylogenetically Widespread and Phenotypically Variable Phenomenon
Source: PLoS One. 2014 Jan 8;9(1):e83259. doi: 10.1371/journal.pone.0083259 (PMC3885428; doi:10.1371/journal.pone.0083259)
Supplement: Table S2 — GenBank accession numbers and sources for DNA sequences utilized in the phylogenetic reconstruction shown in Fig. 2 and Fig. S1. (PDF) [file pone.0083259.s003.pdf]

Table 2. GenBank accession numbers and sources for DNA sequences utilized in this study.

Species	COI	Glyt	myh6	plagl2	rag1	zic1
Abalistes stellatus	JF492756	JX190373	JX190512	JX190636	JX190901	JX191354
Acanthurus nigricans	EF648267	JX190353	JX190496	JX190621	JX190886	JX191342
Aeoliscus strigatus	NA	NA	JX189647	NA	JX189801	JX189030
Albula vulpes	GU225131	NA	JX190390	NA	JX190803	JX191247
Aldrovandia affinis	AP002974.1	NA	JX190392	JX190533	NA	JX191249
Alepisaurus ferox	EU366542	JX190305	JX190451	JX190577	JX190840	JX191299
Ambloplites rupestris	EU524413	JX188709	JQ352815	JQ352860	JF742888	JQ353127
Ameiurus natalis	EU524425	JX190273	JX190416	JX190561	JX190818	NA
Amia calva	AB042952	JX190245	JX190389	JX190531	JX190802	JX191246
Amphiprion ocellaris	NA	NA	NA	NA	AY208631	NA
Anguilla rostrata	EU524441	JX190248	JX190395	NA	NA	JX191252
Antennarius striatus	GU188497	FJ582791	JX190505	JX190629	JX190896	JX191349
Aphredoderus sayanus	JN024806	JX190317	JX190462	JX190586	JX190850	JX191311
Aplochiton taeniatus	NA	JX190292	JX190435	NA	JN232639	NA
Aplodinotus grunniens	EU523923	JX188754	JX189695	JX189381	JX189853	JX189079
Apogon lateralis WR	NA	JX188705	JX189657	JX189339	JX189811	JX189040
Aracana aurita	JQ861007	JX190372	JX190511	JX190635	JX190900	JX191353
Archoplites interruptus	JN024809	JX190346	JQ352816	JQ352861	JF742889	JQ353128
Argentina silus	NA	JX190279	JX190423	NA	JX190822	JX191275
Argyropelecus gigas	EU148084	NA	JX190448	JX190574	JX190837	JX191296
Arrhamphus sclerolepis	NA	NA	JX189640	JX189324	NA	JX189025
Assurger anzac	JN990845	JX190381	JX190521	JX190644	JX190910	JX191364
Astyanax mexicanus	HQ557152	JX190268	JX190411	JX190556	NA	NA
Ateleopus japonicus	AP002916	JX190303	JX190449	JX190575	JX190838	JX191297
Aulostomus maculatus	JQ842017	JX188694	JX189646	JX189329	JX189800	JX189029
Barbourisia rufa	JQ354000	JX190324	JX190468	JX190591	JX190855	JX191314
Bathylaco nigricans	AP009494	JX190277	JX190421	NA	NA	NA
Bathylagus euryops	NA	JX190280	JX190424	NA	NA	JX191276
Bathypterois articolor	NA	JX190307	JX190453	JX190579	JX190842	JX191300
Bathysaurus ferox	EU366547	NA	NA	EU366643	EU366689	EU366734
Benthalbella macropinna	EU366552	NA	NA	EU366648	EU366694	EU366739
Benthosema glaciale	KF768167	NA	NA	NA	KF768155	KF768160
Beryx decadactylus	JF492956	JX188692	JX189644	JX189327	JX189798	NA
Betta splendens	JQ667506	JX188827	JX189768	NA	JX189928	JX189154
Bothus lunatus	JQ842025	JX190361	JX190501	NA	JX190891	NA
Brotula multibarbata	JQ431501	JX188680	JX189630	JX189315	JX189782	JX189013
Callechelys catostoma	KF768168	NA	NA	NA	NA	NA
Callionymus bairdi	JQ840435	JX190380	JX190518	JX190642	JX190908	JX191361
Cantherhines pullus	JQ841489	JX190377	JX190515	NA	JX190904	JX191357
Caranx crysos	GU702378	JX190345	JX190490	JX190615	JX190879	JX191336
Cataetyx lepidogenys	NA	JX190337	JX190481	JX190604	JX190868	JX191326
Centroberyx druzhinini	HQ945941	JX190329	JX190473	JX190596	JX190860	JX191319
Centropomus unidecimalis	JQ365276	JX188711	JX189662	JX189344	JX189816	JX189045
Centropyge flavissima	JQ431559	NA	NA	NA	EF530089	NA
Cephalopholis argus	JX674947	JX188757	JX189698	JX189384	JX189856	JX189082
Chaetodon striatus	JQ840451	JX190349	JX190492	JX190617	JX190882	JX191338
Chanos chanos	HQ654700	JX190263	JX190408	JX190551	JX190809	JX191264
Chaunax suttkusi	NA	JX190368	JX190507	JX190631	JX190898	JX191351
Chelmon rostratus	FJ583134	JX190350	JX190493	JX190618	JX190883	JX191339
Chilomycterus schoepfi	JQ842420	JX188832	JX189773	JX189454	JX189933	JX189158
Chitala chitala	FJ918897	JX190259	JX190405	JX190546	FJ896406	JX191260
Chlorophthalmus agassizi	EU366553	NA	NA	NA	EU366695	EU366740
Chologaster cornuta	HQ557553	JX190318	HQ729508	HQ729624	HQ707706	HQ729595
Chromis cyanea	JQ840457	JX188795	NA	JX189416	JX189896	JX189123
Cirrhitichthys falco	FJ583255	NA	JQ352824	JQ352869	NA	JQ353136
Conger oceanicus	NA	JX190249	JX190396	JX190537	NA	JX191253
Coregonus clupeaformis	EU523959	JX190297	JX190441	JX190568	JX190831	JX191289
Coryphaena hippurus	JQ839745	JX190342	JX190488	JX190612	JX190876	JX191333
Coryphaenoides rupestris	NA	EU002054	EU001915	NA	FJ215233	NA
Cottus carolinae	JN025051	JX188700	JX189652	JX189334	JX189806	JX189035
Cromeria nilotica	AP007275	JX190265	JX190410	JX190553	JX190811	JX191266
Cyttopsis rosea	JQ774524	JX190321	JX190465	JX190588	JX190852	NA
Denticpes clupeoides	AP007276	NA	JX190407	JX190549	DQ912100	NA
Diaphus dumerilii	KF768169	NA	NA	NA	KF768156	KF768161
Diodon holocanthus	GU225199	JX188833	JX189774	JX189455	JX189934	JX189159
Diplophos taenia	EU366584	NA	FJ918866	EU366676	EU366724	EU366768
Dissostichus eleginoides	JN640627	JX188801	JN187040	JX189422	JX189902	JN186822
Echeneis naucrates	JQ841117	JX190343	NA	JX190613	JX190877	JX191334
Echidna rhodochilus	NA	JX190251	JX190397	JX190538	JX190805	NA
Elassoma zonatum	JN025308	JX190386	JQ352848	JQ352893	JQ353047	JQ353156
Eleotris pisonis	AY722157	JX188817	JX189758	JX189440	JX189918	JX189145
Elops saurus	AP004807	JX190247	JX190394	JX190535	FJ896408	JX191250
Enneapterygius gruschkai	HQ168540	NA	NA	NA	HQ168770	NA
Esox lucius	EU524591	JX190284	JX190428	EU002099	JX190825	EU001870
Etheostoma atripinne	HQ557533	JX188739	NA	JX189366	JX189838	JX189065
Eurypharynx pelecanoides	AB046473	JX190254	JX190402	NA	NA	JX191255
Facciolella gilbertii	KF768170	NA	NA	NA	NA	KF768162
Fistularia petimba	JQ365356	NA	JX190482	JX190607	JX190871	NA
Forcipiger flavissimus	JF434972	JX188713	JX189664	JX189346	JX189818	JX189047
Galaxias maculatus	AP004104	JX190294	JX190437	NA	N232643	JX191286
Galaxiella nigrostriata	AP006853	JX190295	JX190438	NA	JN232647	JX191287
Gambusia affinis	JQ842475	JX188691	JX189643	JX189326	JX189797	JX189027
Gasterosteus aculeatus	JQ354102	JX188696	JX189649	JX189331	JX189803	JX189032
Gazza minuta	DQ028007	JX188724	JX189671	JX189353	JX189825	JX189054
Gephyroberyx darwini	HQ945918	JX190334	JX190478	JX190601	JX190865	JX191324
Gigantura indica	EU366557	NA	NA	EU366652	EU366699	EU366744
Gnathonemus petersii	HM880237	JX190258	NA	JX190545	JX190807	JX191259
Gonrynchus greyi	AB054134	JX190264	JX190409	JX190552	JX190810	JX191265
Gymnorhamphichthys petiti	NA	JX190267	NA	JX190555	JX190813	JX191268
Halichoeres bivittatus	JQ840524	JX188785	JX189728	JX189410	JX189885	JX189111
Halieutichthys aculeatus	NA	JX190370	JX190509	JX190633	NA	NA
Halosauropsis macrochir	EU869816	JX190246	JX190393	JX190534	NA	NA
Harpadon marchohir	EU366558	NA	NA	EU366653	EU366700	EU366745
Helostoma temminckii	NA	JX190384	JX190524	JX190647	JX190913	JX191366
Heteroconger hassi	JQ350055	JX190250	NA	NA	NA	JX191254
Heteromycteris japonicus	ABFJ254	JX190365	JX190504	JX190628	JX190895	JX191348
Himantolophus sagamius	GU440342	JX190369	JX190508	JX190632	JX190899	JX191352
Hime japonica	AB047821	NA	NA	EU366641	EU366687	EU366732
Hiodon tergisus	EU524659	JX190257	JX190404	JX190544	NA	JX191258
Histiophryne cryptacantus	GU188513	JX190367	JX190506	JX190630	JX190897	JX191350
Hoplostethus atlanticus	JN580187	JX188693	JX189645	JX189328	JX189799	JX189028
Hypoptychus dybowskii	NC4400	JX190341	JX190487	JX190611	JX190875	JX191332
Ijimaia loppei	NA	JX190304	JX190450	JX190576	JX190839	JX191298
Ipnops sp	EU366560	NA	NA	EU366655	EU366702	EU366747
Kurtus gulliveri	EU381031	JX190339	JX190485	JX190609	JX190873	JX191330
Labrisomus multiporosus	NA	JX188812	JX189752	JX189434	JX189914	JX189139
Lachnolaimus maximus	JQ841240	JX188788	JX189732	JX189411	NA	JX189115
Lactophrys triqueter	JQ861018	JX188835	JX189776	JX189457	JX189936	JX189161
Lampris guttatus	DQ885096	JX190314	NA	JX190584	JX190848	JX191308
Lates niloticus	GU324190	JX190382	JX190522	JX190645	JX190911	NA
Leiognathus equulus	DQ028017	JX188725	JX189672	JX189354	JX189826	JX189055
Lepidogalaxias salamandroides	FJ918925	NA	JX190440	NA	FJ896438	JX191288
Lepidogobius lepidus	NA	NA	JX190519	JX190643	JX190909	JX191362
Lepomis macrochirus	EU524741	JX190347	JX190491	JX190616	JX190880	JX191337
Liopropoma rubre	JQ841244	NA	HQ731360	HQ731341	HQ731318	HQ731189
Liparis mucosus	JQ354182	JX190387	JX190525	NA	JX190915	JX191368
Lophius americanus	EU660715	JX188679	JX189629	JX189314	JX189781	JX189012
Lota lota	HQ961084	JX190320	JX190464	NA	JX190851	JX191313
Luvarus imperialis	AP009161	JX190355	EF536299	EF536261	EF530099	EF533922
Lycodes terraenovae	NA	EF032996	EF032931	EF033022	NA	EF032918
Maccullochella peelii	DQ107940	JX188737	JQ352837	JQ352882	JQ353036	JQ353148
Macropinna microstoma	EU869816	JX190282	JX190426	NA	JX190823	JX191278
Macroramphosus scolopax	JQ775070	NA	JX190483	JX190608	JX190872	JX191329
Mallotus villosus	HQ712650	JX190289	FJ918856	NA	DQ397097	FJ906654
Masturus lanceolatus	AP006239	JX190374	EF536316	EF536277	AY308792	EF533939
Megalops atlanticus	AP004808	NA	NA	JX190536	AY430204	JX191251
Meiacanthus grammistes	NA	JX188809	JX189748	JX189430	JX189910	JX189135
Melamphaes polylepis	NA	JX190325	JX190469	JX190592	JX190856	JX191315
Mene maculata	GU805056	JX190352	JX190495	JX190620	JX190885	JX191341
Micropterus salmoides	HQ557266	JX190348	JQ352819	JQ352864	JF742912	JQ353131
Mola mola	JX438518	JX190375	JX190513	JX190637	JX190902	JX191355
Monocentris japonica	DQ648452	JX190332	JX190476	JX190599	JX190863	JX191322
Monopterus albus	AP002945	JX188698	JQ352812	JQ352857	JQ353018	JQ353124
Monotaxis grandoculis	JQ431907	JX188727	JX189673	JX189356	JX189828	JX189057
Morone chrysops	EU524141	JX188732	JX189678	JX189361	JX189833	JX189061
Muraenichthys	JQ431927	NA	NA	NA	NA	NA
Myrichthys maculosus	NA	JX190252	JX190399	JX190539	NA	NA
Myripristis violacea	HM034239	JX190330	JX190474	JX190597	JX190861	JX191320
Nansenia ardesiaca	AP004106	JX190281	JX190425	NA	NA	JX191277
Naso lituratus	HM034244	JX190354	EF536292	EF536254	EF530092	EF533915
Neochanna_burrowsius	NA	JX190296	JX190439	NA	JN232658	NA
Neonesthes capensis	KF768171	JX190301	JX190445	JX190572	NA	JX191293
Neosalanx jordani	HM151574	HQ916019	NA	NA	HQ916058	HQ916188
Neoscopelus microchir	KF768172	JX190309	JX190456	JX190581	NA	JX191303
Normichthys operosus	FJ918918	NA	FJ918852	NA	FJ896431	FJ906650
Novumbra hubbsi	HQ557339	JX190285	JX190429	NA	AY380546	NA
Ogcocephalus nasutus	NA	JX190371	JX190510	JX190634	NA	NA
Omosudis lowei	EU366566	NA	NA	EU366662	EU366707	EU366750
Ophioblennius atlanticus	FJ583755	JX188810	JX189749	JX189431	JX189911	JX189136
Opsanus pardus	NA	JX190335	JX190479	JX190602	JX190866	NA
Opsariichthys uncirostris	AB218897	JX190274	JX190417	JX190562	JX190819	JX191270
Oryzias latipes	AP004421	Ensembl	Ensembl	Ensembl	Ensembl	Ensembl
Osmerus mordax	JQ354253	JX190287	JX190431	JX190566	JX190827	JX191282
Oxycheilinus celebicus	NA	JX188789	JX189733	NA	JX189888	JX189116
Pachypanchax playfairii	NA	JX190385	NA	NA	JX190914	JX191367
Paraliparis meganchus	NA	JX190388	JX190526	NA	JX190916	JX191369
Paratilapia polleni	AY263886	JX188772	JX189711	JX189397	JX189869	JX189095
Paraulopus oblongus	EU366568	NA	NA	EU366664	EU366709	EU366752
Parupeneus multifasciatus	KF768173	NA	NA	NA	KF768157	KF768163
Percopsis omiscomaycus	EU524269	JX188681	JQ352809	JQ352854	JX189783	JQ353121
Platax teira	HQ149897	NA	EF536282	EF536244	EF530083	EF533905
Plecoglossus altivelis	NC002734	HQ916036	NA	NA	HQ916078	HQ916208
Polymixia japonica	AP002927	JX188676	JX189626	JX189311	JX189778	JX189010
Polyodon spathula	JN028275	NA	NA	JX190530	JX190801	JX191245
Polypterus ornatipinnis	AP004351	JX190244	NA	JX190528	JX190799	JX191243
Porichthys notatus	JQ354298	JX190336	JX190480	JX190603	JX190867	JX191325
Poromitra crassiceps	JQ354300	JX190326	JX190470	JX190593	JX190857	JX191316
Prognathodes aculeatus	NA	JX190351	JX190494	JX190619	JX190884	JX191340
Protosalanx chinensis	JN242658	HQ915999	NA	NA	HQ916039	HQ916166
Psettodes erumei	EF609580	JX190363	JX190503	JX190626	JX190893	JX191347
Pseudochromis fridmani	NA	JX188750	JX189691	JX189377	JX189849	JX189075
Pseudopleuronectes americanus	EU752158	JX188830	JX189771	JX189452	JX189931	JX189157
Pseudotrichonotus altivelis	EU366570	NA	NA	NA	EU366711	EU366754
Ptychochromis grandidieri	AY263878	JX188774	JX189713	JX189399	JX189871	JX189097
Rachycentron canadum	JQ841349	JX190344	JX190489	JX190614	JX190878	JX191335
Ranzania laevis	HQ167729	JX190376	JX190514	JX190638	JX190903	JX191356
Regalecus russelii	NA	JX190315	JX190460	JX190585	NA	JX191309
Retropinna semonis	FJ918924	JX190290	JX190433	NA	JX190829	JX191284
Rheocles wrightae	AY290803	JX188685	JX189633	JX189318	JX189788	JX189020
Rogadius asper	NA	KF139871	KF140114	NA	KF141350	NA
Rondoletia loricata	EU148304	JX190328	JX190472	JX190595	JX190859	JX191318
Salangichthys microdon	FJ205609	HQ916024	NA	NA	HQ916063	HQ916193
Salarias fasciatus	FJ584077	NA	NA	NA	HQ168820	NA
Salvelinus alpinus	JX960951	JX190299	JX190443	JX190570	JX190833	JX191291
Samariscus latus	NA	JX190364	NA	JX190627	JX190894	NA
Sarda sarda	JQ623978	JX188822	JX189763	JX189445	JX189923	JX189150
Sargocentrum cornutum	FJ237590	JX190331	JX190475	JX190598	JX190862	JX191321
Saurida tumbil	KF768174	NA	NA	NA	KF768158	KF768164
Scatophagus argus	JN021250	JX188820	JX189761	JX189443	JX189921	JX189148
Scolopsis bilineata	NA	JX188734	JX189680	JX189363	JX189835	JX189063
Scopelengys tristis	KF768175	JX190310	JX190457	NA	NA	JX191304
Scopelosaurus harryi	EU366572	NA	NA	EU366666	EU366713	EU366756
Sebastes fasciatus	NA	JX188703	JX189655	JX189337	JX189809	JX189038
Sebastolobus alascanus	JQ354498	JX188704	JX189656	JX189338	JX189810	JX189039
Selenotoca multifasciata	DQ107759	JX190356	JX190497	JX190622	JX190887	JX191343
Seriola dumerili	JX261426	JX188707	JX189659	JX189341	JX189813	JX189042
Serranus tigrinus	JQ843057	JX188763	JX189704	JX189390	JX189862	JX189088
Siganus spinus	JQ432159	JX190357	EF536305	EF536267	EF530104	EF533928
Sphyraena barracuda	JQ842711	JX190360	JX190500	JX190625	JX190890	JX191346
Stegastes leucostictus	JQ841016	JX188797	JX189740	JX189418	JX189898	JX189125
Sternoptyx pseudobscura	KF768176	NA	NA	NA	KF768159	KF768165
Stokellia anisodon	NA	JX190291	JX190434	NA	JX190830	JX191285
Stomias atriventer	KF768177	NA	NA	NA	NA	KF768166
Stylephorus chordatus	AB280689	JX190319	JX190463	JX190587	EF094952	JX191312
Sudis atrox	EU366576	NA	NA	EU366670	EU366717	EU366759
Symphurus atricaudus	GU440541	JX190362	JX190502	NA	JX190892	NA
Syngnathus fuscus	JN312447	JX190338	JX190484	NA	NA	NA
Synodus kaianus	EU366578	NA	NA	EU366672	EU366719	EU366761
Tetraodon miurus	NC15361	JX190378	JX190516	JX190639	JX190905	JX191358
Thymallus brevirostris	NA	JX190300	JX190444	JX190571	JX190834	JX191292
Toxotes jaculatrix	HQ654759	JX188767	JX189708	JX189394	JX189866	JX189092
Trachinotus carolinus	JX034020	JX188708	JX189660	JX189342	JX189814	JX189043
Trachipterus arcticus	NA	JX190316	JX190461	NA	JX190849	JX191310
Triacanthodes anomalus	AP009172	JX188836	JX189777	JX189458	JX189937	JX189162
Triacanthus biaculeatus	JF952881	JX190379	JX190517	JX190640	JX190906	JX191359
Umbra limi	EU522452	JX190286	JX190430	NA	JX190826	JX191281
Xentodon cancila	FJ459539	JX188688	JX189636	JX189321	JX189791	JX189023
Xiphias gladius	JQ624016	JX190383	JX190523	JX190646	JX190912	JX191365
Zanclus cornutus	JQ350417	JX190359	JX190499	JX190624	JX190889	JX191345
Zenopsis conchifera	NA	JX190322	JX190466	JX190589	JX190853	NA
Zeus faber	HQ945864	JX190323	JX190467	JX190590	JX190854	NA
